# Psychometric properties of the Positive Aspects of Caregiving Scale for unpaid caregivers of people living with dementia in the Brazilian context: a validation study

**DOI:** 10.1590/1516-3180.2025.3184.06042026

**Published:** 2026-07-10

**Authors:** Sofia Cristina Iost Pavarini, Aline Cristina Martins Gratão, Camila Rafael Ferreira Campos, Diana Quirino Monteiro, Elizabeth Joan Barham, Fabiana de Souza Orlandi, Gabriela Martins, Gustavo Carrijo Barbosa, Keila Cristianne Trindade da Cruz, Larissa Corrêa, Luana Aparecida Rocha, Ludmyla Caroline de Souza Alves, Ana Carolina Ottaviani

**Affiliations:** I Department of Nursing, Department of Gerontology, Universidade Federal de São Carlos (UFSCar), São Carlos (SP), Brazil.; II Department of Nursing, Department of Gerontology, Universidade Federal de São Carlos (UFSCar), São Carlos (SP), Brazil.; III Universidade Federal de São Carlos (UFSCar), São Carlos (SP), Brazil.; IV Universidade Católica de Brasília (UCB), Brasília (DF), Brazil.; V Department of Psychology, Universidade Federal de São Carlos (UFSCar), São Carlos (SP), Brazil.; VI Department of Nursing, Department of Gerontology, Universidade Federal de São Carlos (UFSCar), São Carlos (SP), Brazil.; VII Department of Gerontology, Universidade Federal de São Carlos (UFSCar), São Carlos (SP), Brazil.; VIII Universidade Federal de São Carlos (UFSCar), São Carlos (SP), Brazil.; IX Department of Nursing, Universidade de Brasília (UnB), Brasília (DF), Brazil.; X Universidade Federal de São Carlos (UFSCar), São Carlos (SP), Brazil.; XI Universidade Federal de São Carlos (UFSCar), São Carlos (SP), Brazil.; XII Universidade Federal de São Carlos (UFSCar), São Carlos (SP), Brazil.; XIII Universidade Federal de São Carlos (UFSCar), São Carlos (SP), Brazil.

**Keywords:** Validation study, Caregivers, Dementia, Mental health, Psychometric properties, Family caregivers, Positive aspects of caregiving, Major neurocognitive disorder

## Abstract

**BACKGROUND:**

The Positive Aspects of Caregiving Scale (PAC) assesses the satisfaction and psychosocial benefits perceived by caregivers in the caregiving experience.

**OBJECTIVE:**

To analyze the psychometric properties of the PAC among unpaid caregivers of people living with dementia in the Brazilian context.

**DESIGN AND SETTING:**

Validation study of the instrument conducted online with caregivers from different regions of Brazil.

**METHODS:**

The sample comprised 100 unpaid caregivers of people living with dementia. Sociodemographic characteristics, positive aspects of caregiving, burden, depressive symptoms, anxiety symptoms, and quality of life were assessed using an online form. Evidence of validity based on internal structure was examined using confirmatory factor analysis, reliability testing, and criterion validity analysis.

**RESULTS:**

Most participants were women (95%), daughters of the care recipient (85%), with an average age of 54.5 years, a high educational level, and residence in the Southeast region of Brazil. Depressive symptoms (70%), anxiety (65%), moderate to severe burden (42%), and low quality of life (62%) were prevalent. Factor analysis indicated satisfactory fit for the PAC, and the high correlation between factors supported consideration of a one-dimensional scoring structure. The scale showed high internal consistency (α = 0.89) and significant correlations with burden, anxiety symptoms, depressive symptoms, and quality of life.

**CONCLUSION:**

The PAC showed good psychometric properties in the Brazilian context, supporting its validity and reliability for assessing positive aspects of caregiving among unpaid caregivers of people living with dementia.

## INTRODUCTION

Globally, over 55 million people live with dementia, with nearly 10 million new cases expected annually, making dementia a public health problem and one of the leading causes of disability and dependency.^1^ Most people with dementia are cared for by unpaid caregivers, particularly in low- and middle-income countries.^2^

Studies indicate that unpaid caregivers of people living with dementia often face considerable challenges, including potentially deleterious effects on well-being, such as burden, depression, anxiety, decline in physical capacity, and reduced quality of life.^3,4,5^ However, caregivers may also report positive experiences when caring for family members, and these experiences may play an important role in their well-being and quality of life.^3,6^

Positive aspects of caregiving refer to the gains or satisfaction that caregivers derive from the caregiving experience.^7^ Satisfaction, rewards, perceived gains, competence, meaning, resilience, self-efficacy, personal growth, and a sense of mastery are commonly described as positive aspects in the literature.^6,8,9^

A study involving 1,222 unpaid caregivers of people living with dementia in the United States found that positive aspects of caregiving were inversely associated with depressive and anxiety symptoms. Caregivers who reported higher levels of positive aspects of caregiving had fewer depressive and anxiety symptoms than those with lower levels. Thus, positive aspects of caregiving may make the burden of care more tolerable and may help alleviate caregiver distress over time.^10^

The Positive Aspects of Caregiving (PAC) Scale is widely recognized as an instrument for assessing the psychological benefits of caregiving among caregivers.^6,8^ The PAC was originally developed by Tarlow et al.^7^ in 2004 and initially consisted of 11 items. After exploratory and confirmatory factor analyses, the instrument was reduced to nine items. The scale comprises two factors (self-affirmation and perspective in life) and yields a total score ranging from 9 to 45 points, with higher scores indicating greater positive perception and benefits derived from the caregiving experience. The internal consistency of the overall instrument was α = 0.89.^7^

Although the PAC has been used among unpaid caregivers of people living with dementia in the United States,^7,11^ Europe,^12,13^ and Asia,^14,15,16^ evidence on its use in Brazil remains limited. Recently, the PAC was translated and culturally adapted for use with unpaid caregivers in the Brazilian context, following internationally recommended guidelines for cross-cultural adaptation, including translation, back-translation, expert committee review, and pre-testing.^17^ However, evidence regarding its psychometric properties in Brazil remains limited, reinforcing the need for validation studies to support its use in research and clinical practice.^17^

## OBJECTIVE

To analyze the psychometric properties of the Positive Aspects of Caregiving Scale among unpaid caregivers of people living with dementia in the Brazilian context.

## METHODS

This validation study examined the PAC Scale, which had previously been translated and culturally adapted into Brazilian Portuguese.^17^ This study is part of a larger umbrella project entitled iSupport-Brazil,^18^ which evaluates the implementation and effects of the World Health Organization’s iSupport program, an online psychosocial intervention designed to support unpaid caregivers of people living with dementia^18^. All scientific and ethical guidelines were followed, and the study was approved by the Human Research Ethics Committee of the Universidade Federal de São Carlos (CAAE No. 88157118.0.1001.5504).

The study sample comprised 100 unpaid caregivers of people living with dementia from different regions of Brazil. The sample size was determined according to the recommendations of Hair et al.^19^ based on the number of items in the scale. The inclusion criteria were defined according to the iSupport-Brazil umbrella project and included being at least 18 years of age, self-identifying as an unpaid caregiver of a person living with dementia, having provided care for at least six months, residing in Brazil, and having access to a smartphone, computer, or tablet with internet access.

In addition, only caregivers who scored equal to or above the minimum cut-off in at least one of the following measures were included: general perception of burden (≥ 4), anxiety symptoms (≥ 3), or depressive symptoms (≥ 3). These cut-off points were adopted to ensure the inclusion of caregivers experiencing at least a minimal level of emotional burden or psychological distress, in accordance with the eligibility criteria of the iSupport-Brazil intervention,^18^ which targets caregivers at higher psychosocial risk. Caregivers of institutionalized individuals were excluded.

Participants were recruited through online dissemination linked to the iSupport-Brazil umbrella project.^18^ Interested caregivers completed a pre-registration form and were subsequently invited to complete an online screening questionnaire to assess the eligibility criteria. Caregivers who met the inclusion criteria received a new link to the full data collection form. In this form, they provided digital informed consent and answered questions on sociodemographic and caregiving characteristics, positive aspects of caregiving, burden, depressive and anxiety symptoms, and quality of life. Data were collected online between January and May 2024.

In addition to the PAC, the following variables of interest were assessed:

- **Sociodemographic and caregiving characteristics**: sex (female or male), age (continuous), marital status (with or without partner), ethnicity/skin color (white, brown, black, or yellow), region of the country (Southeast, Northeast, Midwest, South and North), number of years in school (5–8 years, 9–11 years, or ≥ 12 years), relationship to the care recipient (spouse, sibling, grandchild, or other), and diagnosis of the care recipient’s type of dementia (Alzheimer’s disease, vascular dementia, frontal-temporal dementia, Lewy body dementia, or I do not remember/do not know). All information was self-reported by participants through the online questionnaire.
- **Burden:** Burden was assessed using the Zarit Burden Interview (ZBI), which consists of 22 items rated on a Likert scale from 0 to 4 points and measures subjective burden. Scores between 21 and 40 indicate mild burden, scores between 41 and 60 indicate moderate to severe burden, and scores above 61 indicate intense burden.^20,21^
- **Anxiety and depressive symptoms:** Anxiety and depressive symptoms were assessed using the Hospital Anxiety and Depression Scale (HADS), which consists of 14 items, with seven questions for each subscale, rated on a Likert scale from 0 to 3 points. Scores >8 suggest the possible or probable presence of anxiety or depressive symptoms.^22,23^
- **Quality of life:** Caregivers’ quality of life was assessed using the caregiver version of the Quality of Life in Alzheimer’s Disease scale (QoL-AD), which consists of 13 items rated on a Likert scale from 1 to 4. Based on the median score of 32 points, higher values indicate better quality of life^24^˒^25^.

Statistical analyses were performed using SAS software version 9.4, except for confirmatory factor analysis, which was conducted using MPLUS software version 6.12. After data collection, descriptive analyses were calculated, including measures of frequency (relative and absolute), position (minimum, mean, and maximum), and dispersion (standard deviation). To assess the fit of the original models to the data, the following fit indices were used: chi-squared test (*χ* ^2^), Comparative Fit Index (CFI), Tucker-Lewis Index (TLI), Root Mean Square Error of Approximation (RMSEA), and Weighted Root Mean Square Residual (WRMR). The adequacy criteria considered were CFI and TLI (≥ 0.9), RMSEA (< 0.06), and WRMR (< 1), as recommended in the literature.^26,27,28^

Cronbach’s alpha was calculated to assess the internal consistency of the instruments, with values >0.6 considered satisfactory.^29^ Spearman’s correlation coefficient was used to assess the relationship between scores, according to the following criteria: weak correlation (<0.3), moderate correlation (0.3–0.5), and strong correlation (>0.5).^29^ A significance level of 5% was adopted for all analyses.

## RESULTS

Among the 100 unpaid caregivers of people living with dementia, 95% were female, with an average age of 54.5 (*SD* 9.61) years. Most participants were white (56%), living with a partner (56%), had ≥ 12 years of schooling (89%) and living in the Southeast region of Brazil (65%). Most were daughters of the person living with dementia and had provided care for 1–3 years (38%). Among care recipients, 72% had Alzheimer’s disease. Moderate to severe burden (42%), anxiety symptoms (65%), depressive symptoms (70%), and lower quality of life (62%) predominated among caregivers. **Table 1** presents the characteristics of the participants.

**TABLE 1:** Descriptive analysis of participants’ sociodemographic, care, and health characteristics, 2024

Variable	n (%)
Sociodemographic characteristics	
Gender	
*Female*	95 (95%)
*Male*	5 (5.05)
Age	
*18–59 years*	70 (70%)
≥ *60 years*	30 (30%)
Schooling	
*5–8 years*	3 (3%)
*9–11 years*	8 (8%)
≥ *12 years*	89 (89%)
Marital status	
*With a partner*	56 (56%)
*Without a partner*	44 (44%)
Ethnicity	
*White*	56 (56%)
*Brown*	33(33%)
*Black*	10 (10%)
*Yellow*	1 (1%)
Country region	
*Southeast*	65 (65%)
*Northeast*	16 (16%)
*Midwest*	10 (10%)
*South*	5 (5%)
*North*	4 (4%)
**Characteristics of care**	
Degree of kinship	
*Child*	85 (85%)
*Spouse*	9 (9%)
*Sibling*	2 (2%)
*Grandchild*	1 (1%)
*Other*	3 (3%)
Care time	
*Less than a year*	12 (12%)
*1–3 years*	38 (38%)
*4–6 years*	21 (21%)
*7–10 years*	23 (23%)
≥ 11 years	6 (6%)
Type of dementia	
*Alzheimer’s disease*	72 (72%)
*Vascular dementia*	10 (10%)
*Frontal-temporal dementia*	5 (5%)
*Lewy Body dementia*	5 (5%)
*I don’t remember/I don’t know*	16 (16%)
**Health characteristics**	
Burden	
*Absent (≤ 20)*	5 (5%)
*Mild (21–40)*	38 (38%)
*Moderate to severe (41–60)*	42 (42%)
*Intense (≥61)*	15 (15%)
Anxiety symptoms	
*Possible/likely (≥ 8)*	65 (65%)
*No symptoms (≤ 7)*	35 (35%)
Depressive symptoms	
*Possible/likely (≥ 8)*	70 (70%)
*Absence of symptoms (≤ 7)*	30 (30%)
Quality of life	
*Higher levels (≥ 32)*	38 (38%)
*Lower levels (< 32)*	62 (62%)

Analysis of the standardized estimates of factor loadings indicates that the PAC Scale items were adequately represented by factors F1 (self-affirmation) and F2 (perspective in life), showing good factor loadings and high statistical significance. All items showed a strong correlation with their respective factors, suggesting that the factors were well defined. However, the high correlation between F1 and F2 (0.872) exceeded the recommended limit of 0.8–0.85, which may indicate poor discriminant validity. This value suggests that the F1 and F2 factors are not fully distinct and may reflect overlapping constructs. The strong correlation between these factors suggests that a single-factor interpretation may be appropriate to reflect the construct measured by the scale (**Table 2**).

**TABLE 2:** Standardized factor loading estimates for the PAC Scale. São Carlos, 2024

	Estimate	Standard error	p value*
F1 BY			
*PAC1 – Caring has made me feel more useful*	0.761	0.045	< 0.001
*PAC2 – Caring has made me feel good about myself*	0.784	0.043	< 0.001
*PAC3 – Caring has made me feel that someone needs me*	0.794	0.044	< 0.001
*PAC4 – Caring has made me feel valued*	0.853	0.036	< 0.001
*PAC5 – Caring made me feel important*	0.737	0.054	< 0.001
*PAC6 – Caring has made me feel strong and confident*	0.829	0.033	< 0.001
F2 BY			
*PAC7 – Caring has allowed me to value life more*	0.873	0.042	< 0.001
*PAC8 – Caring has enabled me to develop a more positive attitude towards life*	0.842	0.037	< 0.001
*PAC9 – Caring has strengthened my relationships with other people*	0.831	0.044	< 0.001
F1 WITH			
*F2*	0.872	0.039	< 0.001

**Table 3** presents the results of the confirmatory factor analysis conducted to assess the fit of the PAC scale in two models: bifactor and unidimensional models. The CFI and TLI indexes were above 0.9, indicating good model fit. However, the RMSEA value exceeded the recommended reference value of ≤0.06, suggesting a reasonable but not optimal fit. Conversely, the WRMR values were below 1, indicating good fit with the observed data. The two-factor model showed marginally better fit indices than the unidimensional model. Nevertheless, the high correlation between self-affirmation and perspective in life suggests that a one-dimensional scoring structure may also be appropriate for representing the overall construct.

**TABLE 3:** Confirmatory factor analysis of the PAC scale. São Carlos, SP, Brasil, 2024

Adjustment index	Model – two factors	Model – single factor
RMSEA	0.117 (CI 90%: 0.077–0.156)	0.126 (CI 90%: 0.088–0.165)
CFI	0.985	0.983
TLI	0.978	0.974
Chi-square	56.621 (24)	62.168 (24)
WRMR	0.637	0.649

RMSEA, root mean square error of approximation; CI, confidence interval; CFI, Bentler’s comparative fit index; TLI, Tucker–Lewis index; WRMR, weighted root mean square residual.

Regarding internal consistency, the Cronbach’s alpha was 0.89 for the total scale, 0.86 for self-affirmation, and 0.8o for perspective in life. These findings indicate satisfactory reliability for the total scale and its domains, with the total score showing high internal consistency (**Table 4**).

**TABLE 4:** Analysis of the internal consistency of the PAC scale, São Carlos, 2024

Item	Item-total correlation	Alpha if item is deleted
PAC Total		
*PAC1*	0.695	0.9
*PAC2*	0.737	0.897
*PAC3*	0.656	0.903
*PAC4*	0.785	0.894
*PAC5*	0.674	0.902
*PAC6*	0.694	0.9
*PAC7*	0.653	0.903
*PAC8*	0.663	0.903
*PAC9*	0.676	0.902
Self-Affirmation		
*PAC1*	0.711	0.871
*PAC2*	0.737	0.867
*PAC3*	0.658	0.879
*PAC4*	0.827	0.851
*PAC5*	0.707	0.871
*PAC6*	0.615	0.886
Perspective in Life		
*PAC7*	0.691	0.744
*PAC8*	0.723	0.706
*PAC9*	0.623	0.806

Crombach’s Alpha.

The PAC showed significant negative correlations with burden, anxiety symptoms, and depressive symptoms, suggesting that higher PAC scores were associated with lower levels of these indicators. In addition, the scale showed significant positive correlations with quality of life, indicating that higher PAC scores were associated with better quality of life. These results suggest that the scale captures caregivers’ positive experiences in a manner consistent with other relevant and theoretically related constructs (**Table 5**).

**TABLE 5:** Analysis of evidence for the construct validity of the PAC scale, São Carlos, 2024

	PAC – total	Self-affirmation	Perspective in life
Zarit	−0.36 (< 0.01)	−0.32 (< 0.01)	−0.35 (< 0.01)
HADS – Depression	−0.32 (< 0.01)	−0.29 (< 0.01)	−0.36 (< 0.01)
HADS – Anxiety	−0.26 (0.01)	−0.24 (0.01)	−0.26 (< 0.01)
QoL-AD	0.39 (< 0.01)	0.35 (< 0.01)	0.4 (< 0.01)

Spearman’s Correlation: ρ (*p* value).

## DISCUSSION

The results of this study indicate that the PAC Scale has adequate psychometric properties for the Brazilian population of unpaid caregivers of people living with dementia. The participants was predominantly composed of women, daughters, and middle-aged caregivers, most of whom cared for family members with Alzheimer’s disease. This profile reflects the characteristics of unpaid caregivers described in the literature.^30,31^ Most participants had over 12 years of schooling and lived in the Southeast region of Brazil, which may have influenced their perceptions of caregiving and the experiences reported in the PAC, considering that cultural differences may affect the relationships between caregiving experiences and caregiver roles.^14^

The internal consistency analysis showed that the PAC had satisfactory Cronbach’s alpha values for both the total scale and its dimensions, demonstrating adequate reliability. In the original study, in which the scale was applied to 1,299 family caregivers of people with Alzheimer’s disease, Cronbach’s alpha was 0.86 for self-affirmation, 0.80 for perspective in life, and 0.89 for the total scale.^7^ The findings of the present study are consistent with international validation studies conducted in different cultural contexts.^11,12,14^

Confirmatory factor analysis revealed that the scale items were well represented by the original factors, self-affirmation and perspective in life. However, the high correlation between these factors suggests possible conceptual overlap, indicating that the constructs underlying the PAC are strongly interrelated. This finding in the Brazilian context corroborates the structural simplification observed in the original version of the scale,^7^ in which the strong bivariate association justified the use of a single global score.

In the present study, the high inter-factor correlation and high reliability of the total score support the use of a global score, reinforcing the hypothesis of a unidimensional interpretation of the scale. In Japan, Furukawa et al.^15^ also found satisfactory validity and reliability for the PAC, with Cronbach’s alpha values of 0.895 for the total scale and 0.896 and 0.823 for the subscales. Exploratory factor analysis revealed a structure different from the original proposal, with two new factors, namely “Living an enriched life” and “Self-usefulness”, demonstrating cross-cultural variations in the conceptual structure of the scale. Despite these structural differences, the Japanese validation also demonstrated high internal consistency and satisfactory psychometric performance, which is consistent with the findings of the present study and supports the overall robustness of the PAC across cultural contexts.

The possibility of a unidimensional interpretation in the Brazilian version, supported by the high correlation between factors and the reliability of the total score, is also consistent with the PAC validation study conducted in Greece. In that study, which included 247 family caregivers, the PAC showed excellent internal reliability (α = 0.898 for the total scale), with components consistent with those observed in the present study. However, Items 7, giving meaning to life, and 8, learning new skills, although originally attributed only to the perspective in life factor, also showed significant loadings in the self-Affirmation factor, indicating possible ambiguity and interrelationship between domains.^13^ These findings reinforce the notion that the domains assessed by the PAC may represent closely interrelated constructs rather than entirely distinct dimensions.

The need to consider alternative models becomes even more evident when comparing the present findings with the PAC validation study conducted in Singapore.^16^ In a national sample of 1,132 caregivers of individuals with functional limitations, a unidimensional model was initially suggested for the nine-item version. However, difficulties in model fit led to the proposal of a bifactor model, including a general and additional specific factors. High inter-item correlations indicated redundancy, resulting in the exclusion of Item 8 due to its overlap with Item 7 and its stronger correlation with Item 9. Consequently, a shortened version of the scale, the S-PAC, was developed and showed adequate psychometric properties.^16^ These results reinforce the importance of considering alternative models to the traditional bifactor structure, particularly in diverse cultural contexts.

The construct validity of the PAC was supported by its significant correlations with measures of burden, anxiety and depressive symptoms, and quality of life. The negative association between PAC scores and levels of burden, anxiety, and depression suggests that perceiving positive aspects of caregiving may mitigate the emotional impact of the caregiver role. Similarly, the positive correlations between PAC scores and quality of life indicate that caregivers who perceive more benefits from caregiving tend to report better quality of life.

A scoping review that included 230 studies on positive aspects of dementia caregiving confirmed these associations.^6^ The authors identified negative correlations between positive aspects of caregiving and depressive symptoms and burden, as well as positive correlations with well-being variables, such as mental health, quality of life, and life satisfaction.^6^ In addition, a systematic review indicated that higher PAC scores were predicted by factors, such as greater self-efficacy, continuity in the caregiving relationship, spirituality or religiosity, use of professional services, and less discomfort with daily care. Conversely, higher levels of burden and stress were associated with lower PAC scores.^8^

These findings reinforce the relevance of the PAC as a tool for assessing the positive impact of caregiving and highlight the importance of careful cross-cultural adaptation. They also indicate the need for future studies to further investigate the factor structure of the scale in different sociocultural contexts, with particular attention to the possibility of a unidimensional structure that adequately represents the experience of Brazilian unpaid caregivers.

Despite the contributions of this study, some limitations should be considered. The participants comprised mostly of caregivers from the Southeast region, which may limit the generalizability of the findings to other regions of Brazil, particularly given the linguistic variations in Portuguese spoken across the country. In addition, the predominance of highly educated participants may have influenced the understanding and interpretation of the scale. Future studies should consider including participants with more diverse scores on the HADS and Zarit scales to examine the performance of the PAC across different levels of psychological distress and burden.

## CONCLUSION

The PAC showed good psychometric properties in the Brazilian context and represents a valid and reliable tool for assessing positive aspects of caregiving among unpaid caregivers of people living with dementia. However, the high correlation between its factors suggests that future research should consider the possibility of a unidimensional structure that may better reflect the experience of these caregivers. This study contributes robust evidence regarding the applicability of the PAC in Brazil and supports its use in future research and interventions aimed at supporting unpaid caregivers, promoting coping strategies, and improving well-being in this population.

## Data Availability

Data supporting the findings of this study are available upon request from the corresponding author, Ana Carolina Ottaviani.
